# An open dataset of Chinese duration expressions

**DOI:** 10.1038/s41597-025-06016-2

**Published:** 2025-11-03

**Authors:** Si-Qi Zhang, Jia-Wen Niu, Xiaoqian Liu, Xiao-Yang Sui, Li-Lin Rao

**Affiliations:** 1 State Key Laboratory of Cognitive Science and Mental Health, Institute of Psychology, Chinese Academy of Sciences, Beijing, China; 2 Department of Psychology, University of Chinese Academy of Sciences, Beijing, China; 3 School of Sociology and Psychology, Central University of Finance and Economics, Beijing, China

**Keywords:** Human behaviour, Human behaviour

## Abstract

Duration information is essential for understanding and analyzing our world. In textual contexts, duration information is typically conveyed in two formats: numeric (e.g., *1* *hour*) and verbal (e.g., *shortly*). To analyze duration information in text, it is crucial to understand how people map duration expressions to corresponding numerical duration. However, the literature has yet to provide lexicons supporting such conversion. Furthermore, existing databases of time-related expressions often lack information about word frequency – a robust predictor of information processing. This article reports an open dataset of 2,101 Chinese duration expressions, each annotated with its corresponding numerical duration. To obtain high-quality data for word frequency, we obtained the frequency of each duration expression from a large-scale corpus of 10 billion Chinese characters (BLCU Corpus Center (BCC) Corpus) and computed an adjusted frequency for each expression. This dataset provides a valuable resource for research on temporal information in Chinese, facilitating studies in natural language processing, psychology, and linguistics.

## Background & Summary

The digitalization of information has made massive amounts of textual data available in the form of books, news articles, blogs and tweets^1^. Currently, advances in automated text analysis techniques have enabled researchers to efficiently process textual data at scale and extract meaningful information for predicting individual characteristics^2^, revealing organizational phenomena^3^ and understanding culture, social processes and other important aspects of social life^1^. Extracting information from textual data often relies on the establishment of corresponding lexicons^1^. With the development of text analysis techniques, an abundance of lexicons focusing on different domains have been established, such as the WordNet lexicon^4^ for cognitive synonyms, the NRC lexicon^5^ for sentiment, and the Linguistic Inquiry and Word Count (LIWC)^6^, which comprises a wide array of psychosocial constructs. Among the various domains covered in the literature, there has been much interest in time-related information in recent years^7^. Time-related information is important because it is essential to how we interpret, organize, and respond to complex events – ranging from understanding antecedents and consequences in organizational processes^8^ to enabling effective diagnosis and treatment in the medical domain^9^. In everyday life, time-related information is expressed through a variety of linguistic expressions, such as “in 2 weeks”, “in a while”, and “now”. Recent advances in the study of linguistic expressions of time have primarily focused on developing lexicons for time orientations (i.e., words that indicate the past, present or future, such as “when”, “now” and “going to”, e.g., LIWC^6^ and TextMind^10^). However, the literature has yet to provide lexicons on an important aspect of time-related information^11^ – duration – which is crucial for capturing the onset and progression of events in text^11^ and for understanding how humans experience and evaluate events^12^. Furthermore, existing databases of time-related expressions often lack information about word frequency – a robust predictor of information processing^13–15^ and a standard component of lexical databases^16,17^. To address these gaps, the current study aims to construct an open dataset on duration expressions that includes frequency information.

Duration refers to a period of time with known length^18^ and is typically expressed in the form of duration expressions such as “a while” or “1 week”. Duration expressions are pervasive in a wide array of circumstances. In everyday communications, people may make appointments to meet each other “in a while”. When making decisions, the reward for choosing an option may be paid “1 week” later. How people map these expressions to corresponding numerical durations has been of substantial interest to researchers in various disciplines. For example, researchers in the natural language processing community have focused on establishing methods to capture and extract temporal information from natural language texts^19,20^. Furthermore, time series forecasting in domains such as energy, finance and health also requires that time-related data be first transformed into a numerical form before it can be analyzed or used for prediction^21^. Converting duration expressions to their corresponding numerical durations is thus a crucial step when extracting and analyzing time-related information in text.

Duration expressions comprise two subcategories: numeric duration expressions (presented in the form of “number + time unit”, e.g., “1 week”) and verbal duration expressions (e.g., “a while”). The conversion from numeric duration expressions to corresponding numerical durations is well determined; for example, when “day” is the target time unit, the expression “1 week” would be transformed to “7 days”. While recent advances in natural language processing have enabled the automatic identification of numeric duration expressions in text^20,22^, there is currently a lack of established open-access lexicons to support the broader reuse of such expressions in other research contexts. Furthermore, existing studies have yet to provide word frequency information for the numeric duration expressions, which limits their utility for researchers.

Verbal duration expressions are usually ambiguous and thus do not support direct conversion into an equivalent numerical duration. Despite their prevalence in natural language, little is known thus far about how verbal expressions can be converted into their numerical equivalents. This knowledge gap limits the capacity of researchers to analyze temporal information conveyed through ambiguous languages. A similar issue exists in studies on probability expressions, where numerical probability expressions are precise, while verbal probability expressions are usually ambiguous^23^. To examine whether people understand probabilistic opinions expressed by ambiguous verbal expressions, an established method in prior research is to ask participants to estimate the numerical values of verbal probability^24–26^. The descriptive statistics of participants’ estimates (e.g., mean, median, standard deviation and interquartile ranges) can be used to map ambiguous verbal expressions to numerical probabilities. Gaining insights from these studies on verbal probability, the current study aims to obtain numerical equivalents for verbal duration expressions using a similar method and construct a Chinese lexicon that covers both numeric and verbal duration expressions and their corresponding numerical durations.

In summary, this article reports a new open dataset on duration expressions in Chinese. The dataset contains 2,101 duration expressions and their corresponding numerical durations. To aid in further exploration of the database, we also provide the frequency and adjusted frequency of use for each verbal and numeric duration expression.

## Methods

**Fig. 1:**
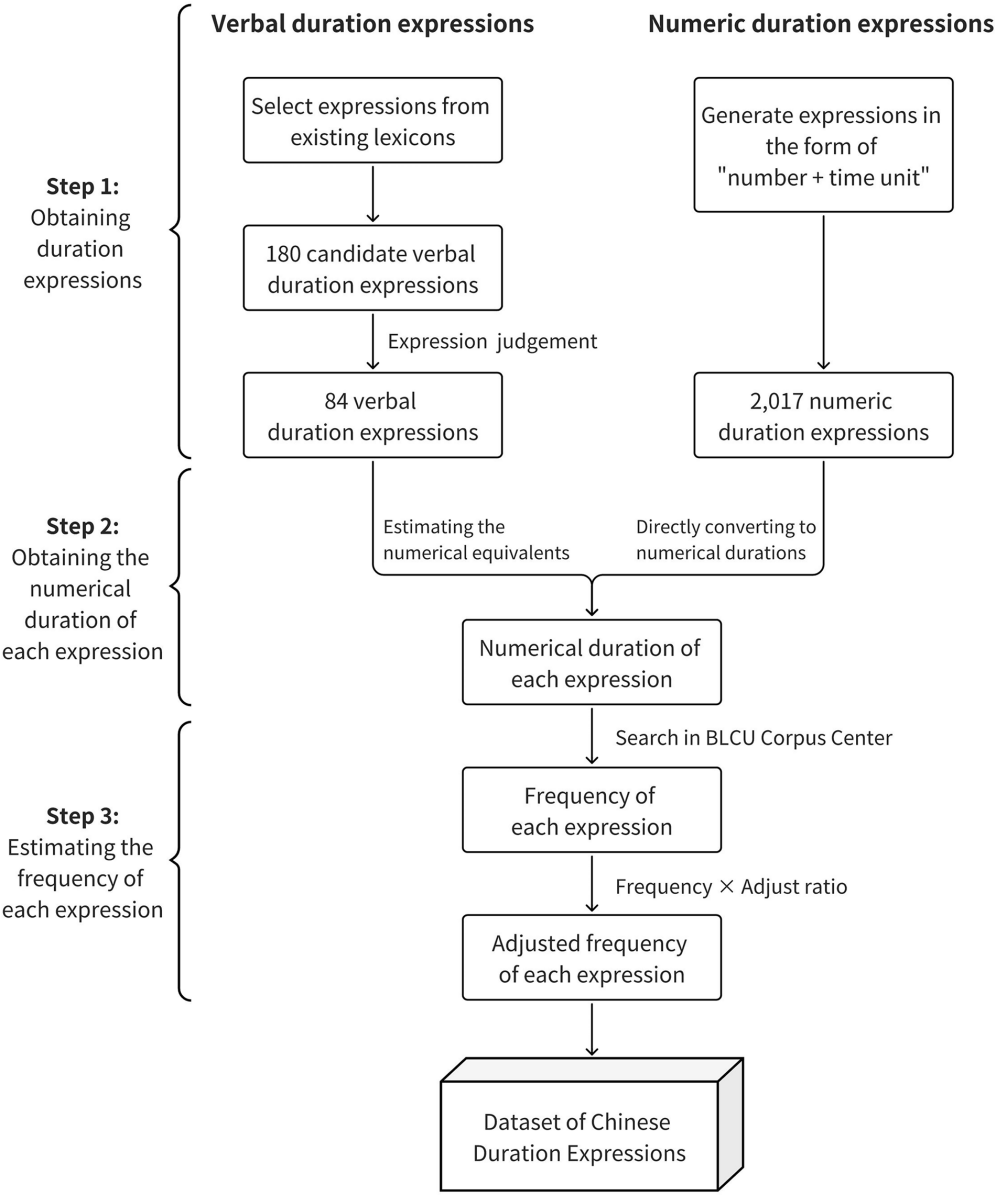
Construction pipeline of the dataset.

The duration expressions contained in the current dataset consists of two parts: verbal duration expressions and numeric duration expressions. For verbal duration expressions, we first identified the candidate expressions from reliable existing lexicons. We then recruited a sample of participants to assess whether each phrase could be used to convey duration information and to estimate the numerical equivalents for each expression. For numeric duration expressions, we generated commonly used expressions ranging from 1 second to 100 years and converted them to corresponding numerical durations. Finally, we obtained the frequency of each verbal and numeric duration expression from the BLCU Corpus Center (BCC) Corpus^27^. Figure 1 depicts the construction pipeline of the dataset. The steps in constructing the dataset are described in detail below. The study protocols were approved by the Institutional Review Board of Institute of Psychology, Chinese Academy of Sciences [No. H23074].

### Obtaining verbal duration expressions and their numerical equivalents

#### Participants

Prior to data collection, all participants received a detailed explanation of the experiments’ purpose, procedure, potential risks and benefits, and gave informed consent through an online consent form to take part in the experiment voluntarily. The consent form informed the participants that (a) the de-identified data may be published in books or academic journals; (b) their anonymized data may be shared via a publicly available data archive; and (c) the researchers are committed to ensuring that all personally identifiable information is strictly protected at all times. All participants are required to actively agree to the terms and confirm their understanding before they could proceed with the questionnaire.

In accordance with previous research^28^, we recruited 55 participants, including 20 males and 35 females, with an average age of 21.62 years (*SD* = 4.74 years). Participants were recruited through advertisements in a local university, and all participants completed two tasks. All participants were paid ¥20 for their involvement. Three participants failed the attention check in Task 1 and thus were excluded. Five additional participants failed the comprehension check in Task 2 and thus were excluded from the analysis of Task 2.

#### Selection of candidate verbal duration expressions

Candidate duration expressions were collected from the following sources: (1) The TextMind lexicon^10^. TextMind consists of two components: (a) the Simplified Chinese version of the LIWC dictionary, and (b) the 5,000 most frequent words collected from the posts of 20,000 active Sina Weibo users. (2) The *Lexicon of Common Words in Contemporary Chinese*^29^. This lexicon contains 56,008 Chinese words that are frequently used in daily life.

Three researchers independently examined both lexicons and selected candidate duration expressions. To avoid the possibility of neglecting any potential expression that could convey duration information, the researchers were requested to select all expressions related to temporal information. In total, we selected 180 candidate verbal duration expressions.

#### Procedure

The in-lab experiment consisted of two tasks conducted in the form of questionnaires on a computer. Task 1 is an expression judgement task. All participants were presented with the 180 candidate verbal duration expressions. For each verbal duration expression, participants made judgements on whether it could be used to express duration (response options: no/unsure/yes). We also presented participants with 5 irrelevant expressions as attention-check questions (i.e., 记者 <journalist>, 国家 <nation> , 棉袄 <padded jacket>, 打扫 <clean> , and 专辑 <album>). Participants who failed to provide correct responses (i.e., no) were excluded from the analysis. The verbal duration expressions were presented in a random order for each participant. Expressions that were judged as “no” (i.e., cannot express duration) by fewer than 20% of the participants were included in the dataset.

Task 2 is a duration estimation task. All participants were asked to estimate the numerical equivalent of each expression that they judged as capable of expressing duration in Task 1. For each expression, participants were first asked to choose an appropriate time unit, including second, minute, hour, day, month, and year, and then to input their estimates. To check whether the participants understood the experimental procedure, we presented all participants with a comprehension check, in which they were asked to estimate the duration of the phrase 两天 <two days> (correct response: 2). Those who failed to provide the correct response were excluded from the analysis. The expressions were presented in a random order for each participant.

#### Data analysis

**Fig. 2:**
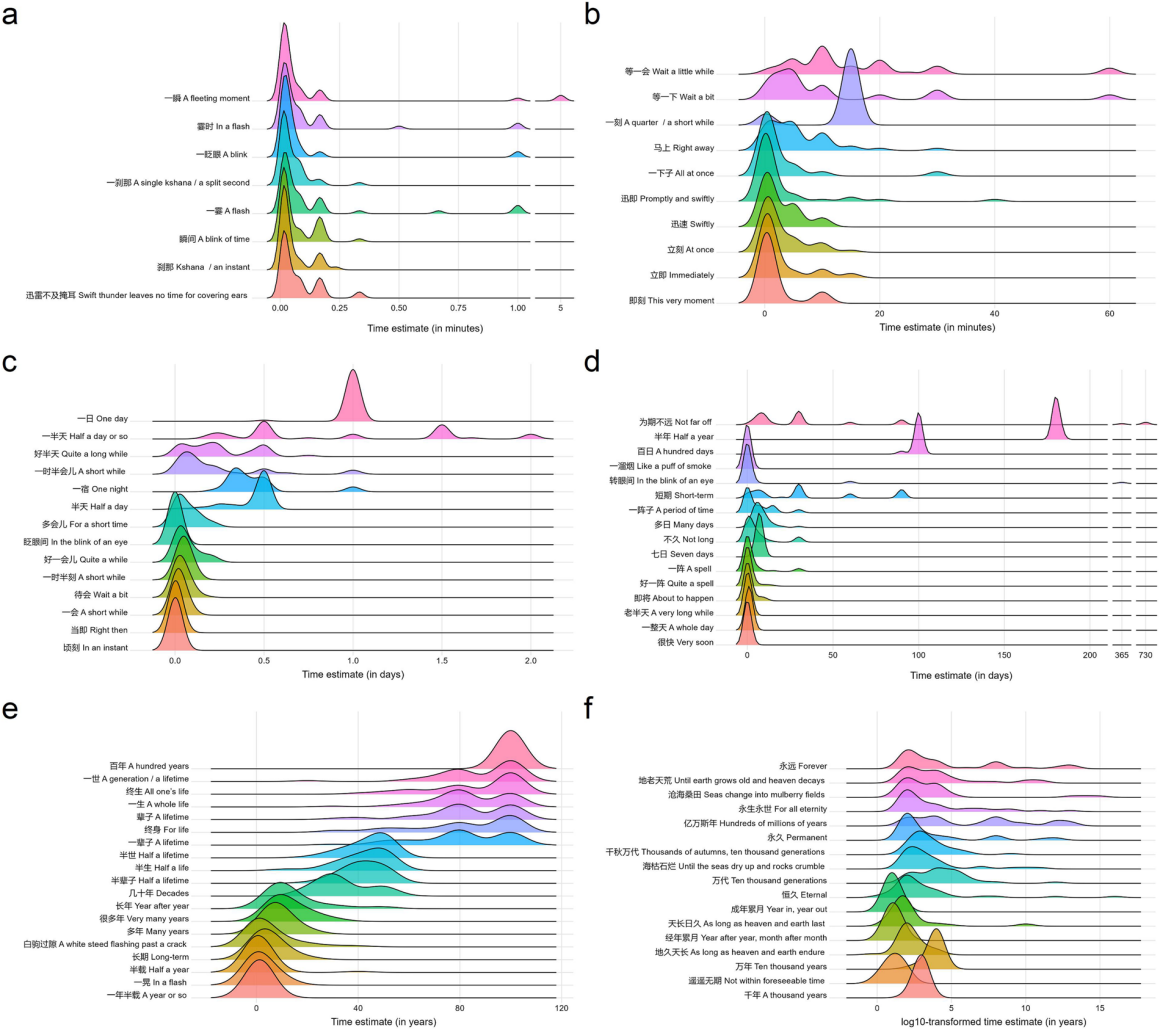
Density plots of duration estimate for verbal duration expressions with a mean estimated duration (**a**) of less than 2 minutes (unit: minutes), (**b**) ranging between 2 minutes and 30 minutes (unit: minutes), (**c**) ranging between 30 minutes and 1 day (unit: days), (**d**) ranging between 1 and 365 days (unit: days), (**e**) ranging between 1 and 100 years (unit: years), and (**f**) greater than 100 years (unit: years). Duration estimates that exceed the 95% quantile were not shown to improve readability of the plot. In **f**, the duration estimates were log10-transformed.

In total, 84 verbal duration expressions were judged as “no” (i.e., cannot express duration) by fewer than 20% of the participants and were included in the dataset. To ensure consistency, we converted all numerical equivalents of duration expressions into days, regardless of the original time units selected by the participants (e.g., years, months). The average estimates from all participants were then used as the numerical equivalent for each duration expression. To provide more detailed information about the distribution of participants’ evaluations of verbal duration expressions, we calculated additional statistical measures for each verbal expression, including standard deviation (*SD*), median (Mdn), interquartile range (IQR), and coefficient of variation (CV). These measures can facilitate further utilization of the database by future researchers. Supplementary Table S1 presents the summary statistics for all verbal duration expressions included in the lexicon. We provide English translations of all verbal duration expressions generated by DeepSeek V3^30^, a leading large language model (LLM) introduced by DeepSeek in 2024^31^. All translations were manually checked by the authors. Figure 2 shows density plots of the duration estimates for each verbal duration expression, calculated by kernel density estimation using the R package *ggridges*^32^.

### Obtaining numeric duration expressions and their corresponding numerical durations

To incorporate numeric durations into the dataset, we generated numeric duration expressions in the form of “number + time unit”, such as 1天 <1 day> and 1年 <1 year> . To capture a broad range of durations encountered in everyday life, our database includes commonly used expressions spanning from 1 second to 100 years. It is worth noting that some numerical durations can be expressed in various ways in Chinese; for example, 2 小时, 2个小时, 两小时, and 两个小时 are all used to express 2 hours. These different expressions were all included in the lexicon. Overall, a total of 2,017 numeric duration expressions were generated and included in the dataset. The corresponding numerical durations of numeric duration expressions can be easily converted by extracting the number and time unit in each expression. For example, the expression 24 小时 <24 hours> is equivalent to a numerical duration of 24 hours and thus 1 day. To ensure consistency, we converted all numeric duration expressions to their corresponding numerical durations in days.

### Estimating the frequency of each verbal and numeric duration expression

Word frequency is a basic and crucial information when conducting text analysis^13,15^. To aid in further exploration of the database, we provide the frequency of use for each verbal and numeric duration expression in the dataset. Word frequency values were obtained from the BLCU Corpus Center (BCC) Corpus (bcc.blcu.edu.cn^27^). The BCC Corpus encompasses a wide range of Chinese texts from diverse sources, including newspapers, literature, academic publications, and social media, totaling approximately 10 billion characters^27^. Given its rich volumes and comprehensive sources, the BCC Corpus is a reliable source for obtaining the frequencies of duration expressions.

Note that some expressions in our lexicon may be used to convey meanings other than duration due to phenomena such as polysemy. For example, 马上 might mean <immediately> or could also mean <on the horse> . To account for this, we analyzed a random sample of fifty occurrences (or all occurrences if fewer than 50 were available) in the BCC Corpus for each duration expression to estimate the proportion of cases where the expression was used to express duration rather than alternative meanings. Since only twenty random occurrences were used to estimate the adjusted frequencies in a previous study on the frequencies of probability phrases^28^, we believed that a random sample of fifty occurrences would provide a stable estimation. For each expression in our lexicon, we calculated the adjusted frequency by multiplying the frequency of each expression and the proportion it was used to express duration. The adjusted frequency offers a more accurate estimate of the frequency with which each expression is used to express duration.

## Data Record

The dataset is available at the repository of the Science Data Bank at 10.57760/sciencedb.28888^33^.

### Data Record 1 – the main dataset

The main dataset is ***lexicon.csv***. This file contains all verbal and numeric duration expressions included in the dataset. The column “expressions” provides the duration expressions. The column “translation” provides English translations for each duration expression, generated by DeepSeek V3 and manually checked by the authors. The column “Type” denotes whether the duration expression is verbal or numeric. The column “num_duration” provides the numerical duration for each duration expression in days. For verbal duration expressions, the numerical equivalent for each expression was determined by averaging the estimates provided by all participants. For numeric expressions, the numerical duration is converted by extracting the number and time unit in each expression. The column “freq” represents the raw frequency of use for each duration expression obtained from the BCC Corpus. The column “adjusted_freq” refer to the adjusted frequency of use for each duration expression.

### Data Record 2 – raw rating data

The subfolder **“1 verbal_expression_processing”** contains the raw data and code for the expression judgement and duration estimation tasks. The **“task1.csv”** file contains the raw data of Task 1 (the expression judgement task). The column “ID” is an identifier distinguishing the study participants. Columns 2–181 refer to participants’ judgements of whether the 180 candidate verbal duration expression could be used to express duration (response options: 1 = yes; 2 = unsure; 3 = no). Columns 182–186 refer to participants’ judgements of whether the five irrelevant expressions could be used to express duration (response options: 1 = yes; 2 = unsure; 3 = no). English translations for all expressions are provided in the second row of the table. The **“task2.csv”** file contains the raw data of Task 2 (the duration estimation task). The column “ID” is an identifier distinguishing the study participants. The second column “两天” <two days> refer to participants’ judgements of the comprehension check (correct response: 2). Columns 3–86 refer to participants’ duration estimates of the 84 verbal duration expressions that were included in the main dataset. A missing value indicates that the participant judged the expression unsuitable for describing duration and thus did not provide a duration estimate. English translations for all expressions are provided in the second row of the table. The **“judgment + estimation.R”** file contains the code for calculating the numeric equivalent and other summary statistics (i.e., standard deviation, median, interquartile range, and coefficient of variation) for each verbal duration expression and the code for reproducing the density plots.

### Data Record 3 – validation analysis

The subfolder **“2 validation”** contains the code for conducting the technical validations (see the Technical Validation section for details).

## Technical Validation

To demonstrate the validity and utility of the current dataset, we provide evidence from the following two perspectives.

First, the construction of the dataset has followed standard practices of lexicon establishment, which ensure that it is rigorously constructed and carefully curated. The duration expressions reported in the dataset were collected from multiple reliable sources (TextMind and the *Lexicon of Common Words in Contemporary Chinese*). The lexicon covers a wide range of time units and incorporates different expressions for the same numeric expression. All verbal expressions in the dataset were validated by human participants, which provided evidence for the quality of the lexicon^34^. The word frequency data was obtained from a large corpus based on diverse sources (the BCC Corpus), which ensures the reliability of the frequency measure^14^. Furthermore, we calculated the adjusted frequency for each expression to better reflect the real-world usage of each expression in the temporal-related context.

Second, by providing an effective tool for the conversion between duration expressions and numerical durations, the current dataset has a high potential for reuse. To demonstrate the validity and utility of the dataset, we tested whether it could be utilized to approximate people’s subjective valuations of temporal delays in a way consistent with predictions from the decision by sampling (DbS) theory^28^. Decision by sampling theory is a theory about how the environment shapes the decision individuals make^35^. According to the DbS, the subjective value of a temporal delay is determined by its relative rank within a random sample of delays retrieved from memory^28^. Specifically, the relative rank of each delay is calculated using the formula *r* = (*R* − 1)/(*N* − 1), where *N* is the total sum of the frequencies of all delays, and *R* is the rank of a particular delay within *N* when sorted in descending order. The distribution of delays stored in memory reflects the distribution in the real world. Stewart *et al*.^28^ collected English numeric duration expressions ranging from 1 day and 1 year and assessed their real-world frequencies through the number of hits produced by Google. Consistent with the predictions of decision by sampling theory, Stewart *et al*.^28^ found that the tendency of people to discount delayed outcomes according to a hyperbolic discounting function arises from the distribution of relative ranks of delays in the real world. To verify the quality of our dataset, we used the delays and frequencies data in our dataset to calculate the relative ranks of delays following Stewart *et al*.^28^, in such a way that high-quality data are necessary to obtain meaningful outcomes.

For comparison with the Stewart *et al*.^28^ study, we included only expressions with numerical durations ranging from 1 day to 1 year for subsequent analysis. Following the approach of Stewart *et al*.^28^, we first conducted an analysis using only numeric duration expressions. To further demonstrate the validity of the verbal duration expression in our dataset, we then extended the analysis to include both numeric and verbal duration expressions in the dataset.

For analysis with only numeric duration expressions, given that some numeric duration expressions may correspond to the same delay, we first accumulated the adjusted frequencies of all numeric duration expressions equivalent to the same delay to determine the frequency of that delay. Next, we sorted delays in descending order and calculated the relative rank of each delay with the formula *r* = (*R* − 1)/(*N* − 1) as mentioned above. The analysis with numeric + verbal duration expressions was conducted under the same process as the analysis with numeric duration expressions, except that we used the mean value of participants’ estimates for each verbal duration expression as its corresponding delay. The relative rank (*r*) was used as the DV of the modeling of temporal discounting.

We fitted the distribution of the relative ranks of delays with eight common forms of temporal discounting function, including the power function *f* (*D*) = *cD*^1−*τ*^*/*(1 − *τ*)^28^, the hyperbola-like function *f* (*D*) = 1/(1 + *kD*)^*s*^ ^36^, the exponential function *f* (*D*) = *e*^−*kD*^ ^37^, the simple hyperbolic function *f* (*D*) = 1/(1 + *kD*)^38^, the hyperboloid function *f* (*D*) = 1/(1 + *kD*^*s*^)^39^, the constant sensitivity function *f* $(D)={e}^{-({kD}{)}^{s}}$^40^, the quasi-hyperbolic function *f* (*D*) = β*e*^−*kD*^, when *D* > 0^41^, and the double exponential function *f* (*D*) = *ae*^−*bD*^ + (1 − *a*)*e*^−*cD*^ ^42,43^. *D* in the functions represents the corresponding delay of each duration expressions, and *c, τ, k*, *s*, β, *a* and *b* are free parameters. Following He *et al*.^44^ and Stewart *et al*.^28^, we also provided the parameter bounds for each function in Table 1. The parameters of each function were estimated using the least-squares method. Then, we evaluated the performance of each model by inspecting model selection criteria, including the Akaike information criterion (AIC) and the Bayesian information criterion (BIC). The AIC and BIC values were calculated using the standard formulas in the case of least squares estimation^45^: AIC = *n* $\log \left(\frac{{SSE}}{n}\right)+2k$; BIC = *n* $\log \left(\frac{SSE}{n}\right)+k\,\log (n)$, where *n* is the sample size, *SSE* is the sum of squared errors, and *k* is the number of free parameters in the model. The analysis was performed using R 4.5.1.

**Table 1:** Model fit statistics for eight common forms of the temporal discounting function.

Function name	Function	Number of parameters	Parameter bounds	Numeric duration expressions			Numeric + verbal duration expressions		
				Estimate	AIC	BIC	Estimate	AIC	BIC
Power function^28^	*f* (*D*) = *cD*^1−*τ*^*/*(1 − *τ*)	2	*c* < 0 *τ* > 1	*c* = −0.18 *τ* = 1.22	−**3362.87**	−**3354.83**	*c* = −0.24 *τ* = 1.29	−3409.18	−3401.08
Hyperbola-like function^36^	*f* (*D*) = 1/(1 + *kD*)^*s*^	2	*k* ≥ 0 *s* ≥ 0	*k* = 1.96 *s* = 0.23	−3338.48	−3330.44	*k* = 1.04 *s* = 0.32	−**3440.01**	−**3431.91**
Exponential function^37^	*f* (*D*) = *e*^−*kD*^	1	*k* ≥ 0	*k* = 0.01	−1306.31	−1302.29	*k* = 0.02	−1451.36	−1447.31
Simple hyperbolic function^38^	*f* (*D*) = 1/(1 + *kD*)	1	*k* ≥ 0	*k* = 0.03	−1613.81	−1609.79	*k* = 0.05	−1828.41	−1824.36
Hyperboloid function^39^	*f* (*D*) = 1/(1 + *kD*^*s*^)	2	*k* ≥ 0 *s* ≥ 0	*k* = 0.36 *s* = 0.40	−3032.07	−3024.03	*k* = 0.32 *s* = 0.50	−3147.68	−3139.58
Constant sensitivity function^40^	*f* $(D)={e}^{-({kD}{)}^{s}}$	2	*k* ≥ 0 *s* ≥ 0	*k* = 0.02 *s* = 0.26	−2801.42	−2793.38	*k* = 0.03 *s* = 0.31	−2840.85	−2832.74
Quasi-hyperbolic function^41^	*f* (*D*) = β*e*^−*kD*^, when *D* > 0	2	0 ≤ β ≤ 1 *k* ≥ 0	β = 0.55 *k* = 0.004	−1991.13	−1983.10	β = 0.57 *k* = 0.006	−2015.57	−2007.47
Double exponential function^42,43^	*f* (*D*) = *ae*^−*bD*^ + (1 − *a*)*e*^−*cD*^	3	0 ≤ *a* ≤ 1 *b* ≥ 0 *c* ≥ 0	*a* = 0.37 *b* = 0.002 *c* = 0.31	−2811.36	−2799.30	*a* = 0.33 *b* = 0.003 *c* = 0.27	−3154.00	−3141.85

### Results for distribution based on numeric duration expressions

**Fig. 3:**
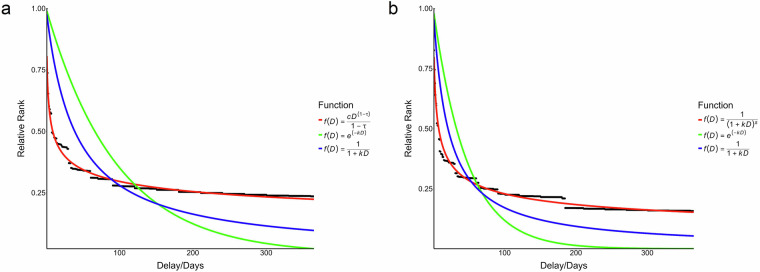
The distribution of relative ranks of durations. The curved lines represent alternative forms of the temporal discounting function fit to the data, including the best-fitting function, the exponential function, and the simple hyperbolic function. (**a**) The results of model fitting based on numeric duration expression data. (**b**) The results of model fitting based on data from both numeric and verbal duration expressions.

We first performed model fitting on the distribution of the relative ranks of delays based on numeric duration expressions. The model fitting results are presented in Table 1. The results showed that the power function *f* (*D*) = *cD*^1−*τ*^*/*(1 − *τ*) provided the best fit (AIC = −3362.87, BIC = −3354.83). Figure 3a illustrates the fit of the best-fitting function as well as the classical exponential and simple hyperbolic functions.

### Results for distribution based on all duration expressions

The model fitting results for the distribution based on all duration expressions are presented in Table 1. The hyperbola-like function *f* (*D*) = 1/(1 + *kD*)^*s*^ provided the best fit (AIC = −3440.01, BIC = −3431.91) to the distribution (see Figure 3b).

In conclusion, with regard to the analysis using only numeric duration expressions, we found that the best-fitting function was the power function (*f* (*D*) = *cD*^1−τ^/(1 − τ)), which is consistent with previous research^28^. This indicates that the current dataset can successfully capture the distributional properties of temporal delays, providing evidence for its validity.

With regard to the analysis incorporating both numeric and verbal duration expressions from the dataset, we found that the hyperbola-like function (*f* (*D*) = 1/ (1 + *kD*)^*s*^) provided the best fit. This pattern suggests a faster decline in the subjective value for shorter delays and a slower decline for longer delays when both numeric and verbal duration expressions are considered. Notably, these findings were consistent with previous research showing that the hyperbola-like function can better describe people’s actual temporal discounting behavior than can other functions^46–48^. The convergence between our results and prior studies suggests that incorporating verbal duration expressions may provide a better proxy for the real-world distribution of temporal delays than relying on numeric duration expressions alone. Therefore, these results not only support the validity of our dataset but also highlight the advantage of incorporating verbal duration expressions in the lexicon.

## Usage Notes

This dataset is intended for researchers interested in investigating time-related textual data. The dataset can be applied to a range of analyses, including but not limited to:

1. The automatic extraction and conversion of duration expressions in natural language processing studies. While prior studies have provided tools for extracting and identifying duration expressions from text^20,22^, these studies have largely focused on numeric duration expressions, for which the conversion to the corresponding numerical duration is precise and well determined. However, people’s everyday communication about time comprises not only precise numeric duration expressions but also a large variety of ambiguous verbal duration expressions^49^. By constructing an annotated lexicon that covers verbal duration expressions, our dataset provides support for studying ambiguous language on duration and thus expands the scope of analysis in time-related textual data.
2. Investigating word frequency effect in temporal expressions. Word frequency is a central variable in many psycholinguistic research and plays a key role in explaining individuals’ visual word recognition^50^. Word frequency effect, a widely studied phenomenon in the past few decades, refers to a tendency that frequently-occurred words are processed easier and more efficiently than low-frequency words^14^. By utilizing the current dataset, researchers can investigate individuals’ processing of temporal information during a lexical decision or naming task. Potential research questions include, but are not limited to: (1) individual and group differences in the processing of time-related information, and (2) the influence of temporal expression processing on real-life behaviors such as temporal discounting.
3. Selection and designation of research materials in behavioral experiments. Many studies in psychology and behavioral sciences, such as those on intertemporal choices and goal pursuit, incorporate temporal information as a key component of their stimuli. The current dataset provides numerical duration estimates and word frequency information for each duration expression, allowing researchers to select and construct materials that meet the desired experimental requirements, and to systematically control for linguistic features such as frequency of use or familiarity. Notably, the verbal duration expressions in our dataset can be used to describe future outcomes with ambiguous or imprecise delays, which have received growing attention in recent years (e.g., “You will get $30 *later*”^51^).

## Supplementary information

Oversized Supplementary Table 1

## Data Availability

The data is accessible without restrictions at the repository of the Science Data Bank at 10.57760/sciencedb.28888^33^.

## Code availability

The code for data preprocessing, analysis and technical validation are available at 10.57760/sciencedb.28888^33^.
